# Proteomic analysis of zoledronic-acid resistant prostate cancer cells unveils novel pathways characterizing an invasive phenotype

**DOI:** 10.18632/oncotarget.2694

**Published:** 2014-11-24

**Authors:** Maria Rita Milone, Biagio Pucci, Katia Bifulco, Federica Iannelli, Rita Lombardi, Ciardiello Chiara, Francesca Bruzzese, Maria Vincenza Carriero, Alfredo Budillon

**Affiliations:** ^1^ Centro Ricerche Oncologiche Mercogliano, Istituto Nazionale Tumori Fondazione G. Pascale - IRCCS, Naples, Italy; ^2^ Experimental Pharmacology Unit, Istituto Nazionale Tumori Fondazione G. Pascale - IRCCS, Naples, Italy; ^3^ Neoplastic Progression Unit, Istituto Nazionale Tumori Fondazione G. Pascale - IRCCS, Naples, Italy

**Keywords:** prostate cancer, zoledronic acid, cytoskeleton organization, αv integrin, urokinase receptor (uPAR)

## Abstract

Proteomic analysis identified differentially expressed proteins between zoledronic acid-resistant and aggressive DU145R80 prostate cancer (PCa) cells and their parental DU145 cells. Ingenuity Pathway Analysis (IPA) showed a strong relationship between the identified proteins within a network associated with cancer and with homogeneous cellular functions prevalently related with regulation of cell organization, movement and consistent with the smaller and reduced cell-cell contact morphology of DU145R80 cells. The identified proteins correlated in publically available human PCa genomic data with increased tumor expression and aggressiveness. DU145R80 exhibit also a clear increase of alpha-v-(αv) integrin, and of urokinase receptor (uPAR), both included within the same network of the identified proteins. Interestingly, the actin-rich structures localized at the cell periphery of DU145R80 cells are rich of Filamin A, one of the identified proteins and uPAR which, in turn, co-localizes with αv-integrin, in podosomes and/or invadopodia. Notably, the invasive feature of DU145R80 may be prevented by blocking anti-αv antibody. Overall, we unveil a signaling network that physically links the interior of the nucleus via the cytoskeleton to the extracellular matrix and that could dictate PCa aggressiveness suggesting novel potential prognostic markers and therapeutic targets for PCa patients.

## INTRODUCTION

Prostate cancer (PCa) is the most commonly diagnosed male cancer in the developed world [1] and a leading cause of cancer-related morbidity and mortality in men worldwide. Although hormonal therapy controls tumor growth for some time, patients eventually become androgen refractory, leading to tumor progression and metastasis, primarily to the bone. While the results of chemotherapy using docetaxel are successful in some patients with castration-resistant PCa (CRPC), others develop chemoresistance, and clinical trials with second-line chemotherapy have been disappointing. Recently, insight into the molecular mechanisms of androgen resistance has led to the development and approval of alternative novel hormonal agents such as abiraterone and enzalutamide [2]. In addition, three other new agents have recently been approved for the treatment of PCa, including the first cancer vaccine Sipuleucil T, the chemotherapeutic agent cabazitaxel, and the radiopharmaceutical radium-223. However, despite continuing efforts in developing novel therapeutic approaches, the overall survival for castration-resistant metastatic disease remains poor [2]. Hence, there is a need to better understand the biology of the disease to develop more effective agents.

Current clinical strategies for evaluating prognosis in PCa at the time of diagnosis include the determination of anatomical extent, histologic grade (Gleason score) and serum levels of prostate-specific antigen (PSA). Although these approaches are clear and important prognostic factors to guide treatment in various clinical contexts, PCa is a heterogeneous disease. Moreover, definitive molecular markers that are able to predict prognosis are not known at present. Thus, identifying the primary involvement of specific pathways could maximize the benefit from targeted therapies and help to define predictive biomarkers in a context of an individualized treatment approach.

Dysregulation of proteins involved in cytoskeleton organization and/or facilitating binding to extracellular matrix has been associated with an increase of cell ability to spread and the acquisition of resistance to antitumor treatments in several tumors, including prostate cancer [3].

Nitrogen-containing bisphosphonates (N-BPs), such as zoledronic acid (ZOL), inhibit osteoclast-mediated bone resorption and are used in clinical practice to reduce skeletal complications and pain related with bone metastasis of several neoplasms including PCa [4]. Accumulating evidence in both preclinical and clinical studies indicate that ZOL might have also an anticancer activity [5–7].

We have recently selected for the first time a ZOL-resistant PCa cell line DU145R80 that exhibits a more aggressive phenotype with increased invasive capability and epithelial-to-mesenchymal transition (EMT) compared to parental DU145 cells [8].

In the present study, to further investigate the mechanism by which cells acquire ZOL-resistance and invasive capability, we took advantage of the highly sensitive two-dimensional differential in gel electrophoresis (2-DE DIGE) to compare cellular extracts from DU145R80 and DU145 cell lines. Applying 2-DE DIGE with dedicated software analysis and successive liquid chromatography (LC)-tandem mass spectrometry (MS/MS) allowed the identification of several differentially expressed proteins. Pathway analysis of experimental data emphasized relevant networks that physically link the interior of the nucleus via the cytoskeleton to the extracellular matrix. Accordingly, the majority of the identified differentially expressed proteins are involved in the regulation of cell organization and movement and are consistent with the smaller and reduced cell-cell contact morphology of DU145R80 cells, most likely reflecting the reorganization of the F-actin cytoskeleton. DU145R80 exhibit also a clear increase of alpha-v-(αv) containing integrins, and of urokinase receptor (uPAR), both included within the same network of the identified proteins. Co-localization and additional functional experiments confirmed that, unlike DU145 cells, DU145R80 cells display actin-rich structures localized at the cell periphery where αv-integrin activation, probably through uPAR, triggers the acquisition of an invasive phenotype. Interestingly, by analyzing publically available cancer microarray datasets, we also found that all the identified protein alterations were associated with PCa compared to normal prostate tissue and were also correlated with aggressive PCa.

## RESULTS

### Comparative proteomic analysis of DU145 and ZOL-resistant derived subline DU145R80

**Figure 1:**
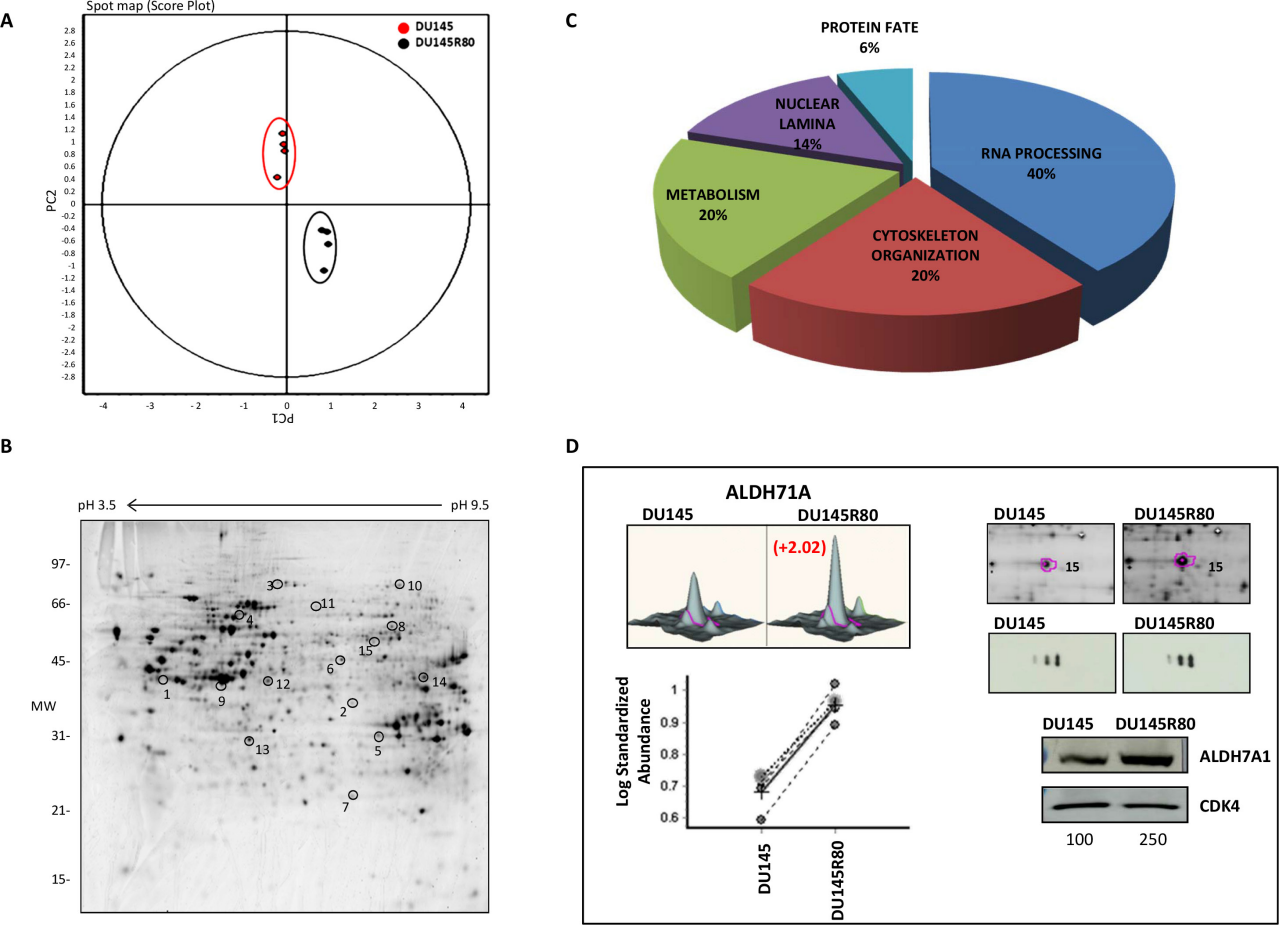
PCA plot, spot map, functional distribution of identified protein and validation by 1-D and 2-D Western blot of ALDH7A1 **(A)** Unsupervised multivariate analysis of the 2-DE DIGE results shows that the eight spot maps clearly clustered into two groups corresponding to DU145 (circled in red) or DU145R80 (circled in black). The PCA plot also shows a good experimental reproducibility as demonstrated by the closely relation between the four biological replicates (DU145 red circles; DU145R80 black circles). To be included in the analysis, protein spots had to be found in 80% of the spot maps and display an expression variation of at least 1.4-fold with *t-Student* test (*p* < 0.05). **(B)** Representative DeepPurple^TM^ stained spot map image of DU145R80. All the detected differences existing between DU145 and DU145R80 cells are visualized by circles. For MS-identified protein spots, the spot numbers match those listed in Table 1. **(C)** Pie chart of functional distribution of proteins identified by 2-DE DIGE/MS. **(D)** The upper-left panels showed the 3D view of the 2-DE DIGE quantification for spot of interest (ALDH7A1). The corresponding between the log of standard abundance of the spot of interest (y-axis) for the two different cell lines (x-axis) on the four replicates are also shown in lower-left panel. Representative 2-DE DIGE gels images are shown on the upper-right panel. The spots of interest corresponding to the ALDH7A1 protein are circled and the spot number match those listed in Table1. The 2D and 1D Western blot results are shown below. Protein lysates from DU145R80 and DU145 cell lines, obtained after 48 h of cell culture, were immunostained with anti-ALDH7A1 antibody. For 1D Western blot CDK4 ensured equal loading of sample in each lane. The quantization of the bands was obtained using the software ImageQuantTL and the value relative to each band normalized on loading control is reported.

DU145R80 were selected from DU145 cells by stepwise exposure to increasing concentrations of ZOL and showed the acquisition of a more aggressive phenotype compared with parental cells [8]. To characterize proteins putatively involved in the resistance to ZOL and in the increased aggressiveness, we analyzed protein expression from DU145R80 and DU145 cell lines after 48 h of cell culture by a 2-DE DIGE proteomics approach. A scheme of the procedure is shown in supplemental Figure S1. In detail, for each condition, biological replicates (performed in quadruplicate) were obtained and reverse-labeled by the fluorescent cyanine dyes Cy3 and Cy5. For the internal standard, which is generated from an equal combination of all the samples tested in the same experiment, the Cy2 dye was used, leading to a proper quantitative comparison of proteomic variations with statistical confidence. In total, approximately 1,200 protein spots were constantly detected in each gel, quantified, normalized and inter-gel-matched. To examine the differences existing between the DU145R80 and DU145 cell lines and to corroborate the biological validity of the biological variation results, acquired data were processed in an unsupervised manner using different multivariate analysis methods according to the DeCyder 2D 7.1 EDA module. Protein spots included in the analysis were those present in 80% of the spot maps and with expression variation of at least ± 1.4-fold at the 95th confidence level (Student's t test *p* < 0.05). Principal component analysis (PCA) showed distinct expression profiles between DU145R80 and DU145 cell lines as well as a consistent reproducibility between the biological quadruplicates (Figure 1A). According to our statistics, 21 spots detected were differentially expressed in DU145R80 compared to DU145 (Figure 1B).

LC-MS/MS was applied for protein identification, and 15 of the 21 protein spots whose levels changed were successfully identified (9 up-regulated and 6 down-regulated in DU145R80 compared with DU145). For each identification, the MASCOT search results are detailed in Table 1 as follows: number of experimental-measured peptide masses matching the theoretical ones obtained from Swiss-Prot/TrEMBL entries, percentage of the protein sequence covered by the matching peptides and probabilistic score. In Table 1, proteins were also specified by fold change and numbers (spot no.), correlating to corresponding spots in Figure 1B. The identified proteins were classified, according to their biological activities into five functional classes. As depicted in the pie chart (Figure 1C), 20% are involved in metabolism including phosphoglycerate kinase 1 (PGK1), Glyceraldehyde-3-phosphate dehydrogenase (GAPDH) and Alpha-aminoadipic semialdehyde dehydrogenase (ALDH7A1); 20% are involved in regulating cytoskeleton reorganization, including Actin cytoplasmic1 (ACTB), Annexin A1 (ANXA1) and Filamin A (FLNA); 6% are involved in protein fate, including Proteasome subunit alpha type6 (PSMA6); 14% are components of the nuclear lamina such as Lamin A/C (LMNA) and LaminB2 (LMNB2); and, finally, 40% consist of proteins involved in RNA processing, including RNA-binding protein4 (RBM4), Elongation Factor2 (EF2), Elongation Factor 1gamma (eEF1γ), ATP-dependent RNA helicase (DDX1), Cleavage Stimulation Factor Subunit 1 (CSTF1) and 60S Acidic Ribosomal Protein P0 (RPLP0).

**Table 1:** Differentially expressed proteins identified by mass spectrometry

Spot no.^a^	Master Spot no.^b^	Spot name^c^	Description	AC^d^	Theoretical pI/Mr (kDa)	Mascot search results			Fold change^e^ DU145R80/DU145
						No. Of matched peptides	Sequencecoverage (%)	Score	
**RNA processing**									
1	1912	RBPM4	RNA-binding protein 4	Q9BYF3	6.61/40.68	11	39	527	−2.80**
3	2037	EF2	Elongation factor -2	P13639	6.41/96.24	7(reg. N-term)	8	232	−1.41**
6	1501	eEF1γ	Elongation factor - 1gamma	P26641	6.25/50.42	4	13	191	1.54*
10	352	DDx1	ATP-dependent RNA helicase DDX1	Q92499	6.81/83.34	21	38	814	2.03*
12	1667	CSTF1	Cleavage stimulation factor subunit 1	Q05048	6.12/49.12	2	8	106	−1.70*
13	2074	RPL0	60 S acidic ribosomal protein P0	P05388	5.71/34.42	3	20	143	1.44*
**Metabolism**									
14	2168	PGK1	Phosphoglycerate kinase1	P00558	8.30/44.98	19	57	920	1.83*
15	1511	ALDH7A1	Alpha-aminoadipicsemialdehyde dehydrogenase	P49419	6.24/55.84	22	53	1127	2.01*
5	2349	GAPDH	Glyceraldehyde-3-phosphate dehydrogenase	P04406	8.16/36.20	12	46	534	1.50*
**Cytoskeleton Organization**									
2	2389	ANXA1	Annexin A1	P04083	6.57/38.91	13	51	677	1.54*
9	1634	ACTB	Actin, cytoplasmic 1	P60709	5.29/42.05	19	56	953	−1.85*
11	635	FLNA	Filamin A	P21333	5.93/83.39	20	37	851	−1.51*
**Nuclear lamina**									
4	695	LMNB2	Lamin B2	Q03252	5.29/67.76	20	33	868	2.32**
8	1259	LMNA	Lamin A/C	P02545-2	6.40/65.15	18	33	742	1.70*
**Protein fate**									
7	3211	PSMA6	Proteasome subunit alpha type-6	P60900	6.34/27.83	6	33	301	−1.40*

a Spot numbers match those present in Figure 1B.

b Master spot numbers.

c Protein acrostic names according UniProtKB.

d UniProtKB Accession Numbers

e Average ratio between the DU145R80 and DU145 cells are reported and significant values indicated (≤ 1.4 or ≥ 1.4; *p < 0.05; **p < 0.01)

**Figure 2:**
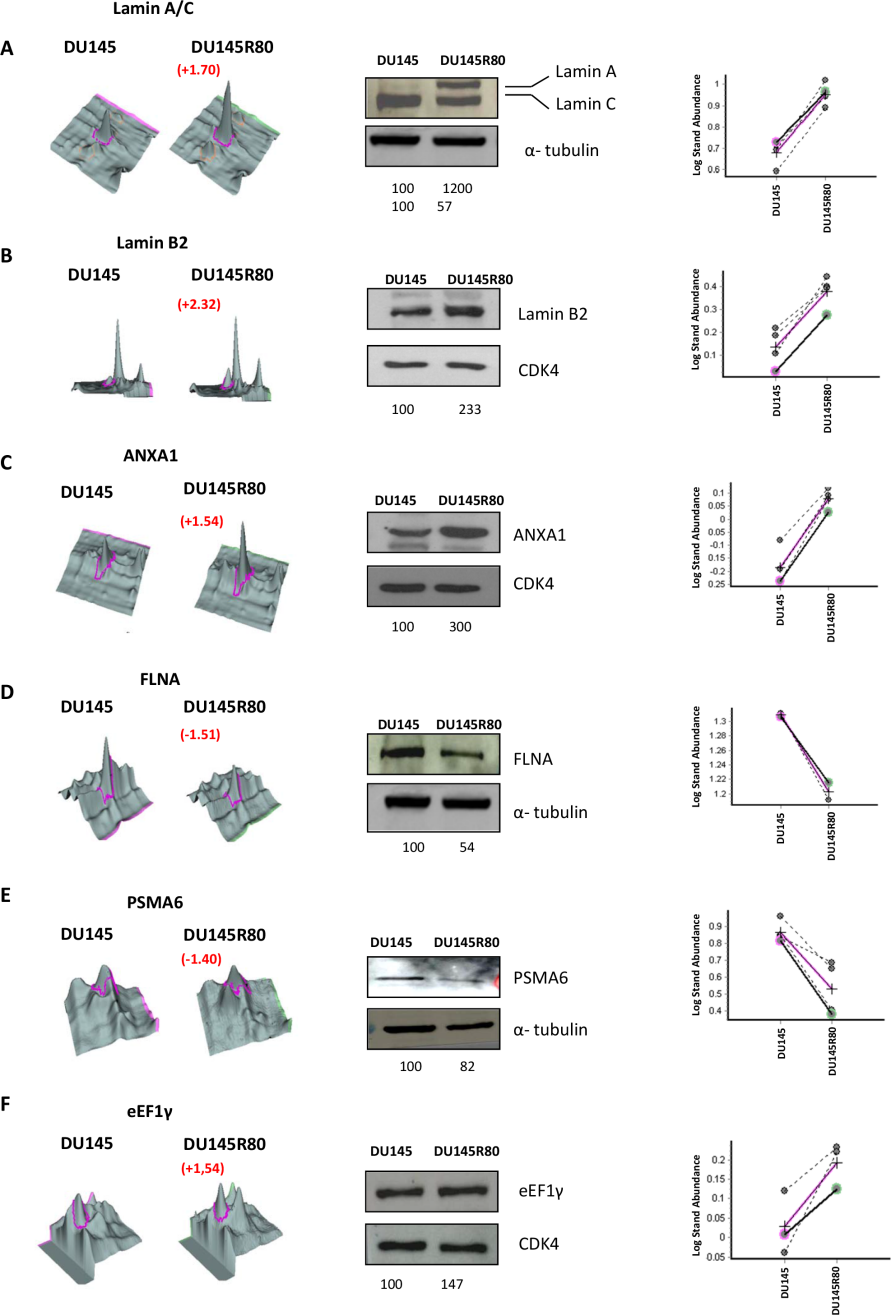
Validation by 1-D Western blot of protein identified as differentially expressed in the 2-DE DIGE/MS analysis In the middle of each validation images 1D Western blot experiment was showed. Protein lysate from DU145R80 and DU145 were immunostained with anti-LAMIN A/C **(A)**; anti-LAMIN B2 **(B)**; anti-AXNA1 **(C)**; anti-FLNA **(D)**, anti-PSMA6 **(E)** and anti-eEF1γ antibodies **(F)**. CDK4 or α-tubulin immunoblotting ensured equal loading of sample in each lane. The quantization of the bands was obtained using the software ImageQuantTL. Below each image the value relative to each band normalized on loading control is reported. On the left of each images the 2-DE DIGE quantification for spots of interest is reported in a 3D view that shows significant difference in protein expression. On the right the corresponding between log of standard abundance of the spot of interest (y-axis) for the two different cell lines (x-axis) on the four replicates are shown.

To validate the 2-DE DIGE-MS/MS-obtained results, as well as to further evaluate the nature and importance of some of the identified proteins that changed expression between DU145R80 and DU145 cells lines, 1-D and 2-D immunoblotting analyses were performed. Proteins with identified expression changes were selected for immunoblotting according to their known or supposed correlation with PCa or with drug resistance based on available literature, and at least one protein for each functional class was analyzed (Figures1D and 2). Western blotting analyses confirmed the 2-DE DIGE findings reported in Figure 1D and 2A-F as spot quantification and as standard abundance.

ALDH7A1 validation was performed by both 1-D and 2-D Western blotting (Figure 1D). Interestingly, 2-D immunoblotting highlighted the up-regulation of three different isoforms of the protein, most likely derived by different splicing sites (http://www.uniprot.org/uniprot/P49419), although we cannot exclude the possibility that they may represent post-translational modifications of the protein. LMNA/C antibody recognized two isoforms: Lamin C and Lamin A, which differ for 6 amino acids at the C terminus. A clear up-regulation was observed only for the isoform protein species A (Figure 2A).

Those proteins validated by immunoblotting were also evaluated for mRNA expression (Figure S2). A clear correlation between protein and mRNA expression was demonstrated only for FLNA, ANXA1 and ALDH7A1. Conversely, PSMA6, eEF1γ, and LMNA mRNA expression apparently are not modulated. LMNB2 mRNA expression was down-regulated while its protein expression was clearly up-regulated in DU145R80 cells compared to DU145. The discrepancy between mRNA and protein expression could indicate either post-transcriptional modulation of those proteins and/or the activation of a feedback inhibitory loop on mRNA expression secondary to protein regulation.

### The biological functions of the identified proteins and morphological analysis confirmed the invasive phenotype of DU145R80 cells

To further characterize the biological functions and the pathways involved in the regulation of the identified fifteen differentially expressed proteins, we used Ingenuity Pathway Analysis (IPA) software. The first analysis was obtained looking for both direct (proteins that make physical contact with one another or induce direct chemical modifications) and indirect interactions. In this way, the most relevant associated network function (score 28) was “Cancer” and included approximately 80% of the identified proteins that closely cluster together, corroborating the biological reliability of the 2-DE DIGE-produced data (Figure S3A). The second analysis included only direct interactions between the identified proteins and selected as the most relevant associated network functions (score 25) “Cellular Assembly and Organization, Cell cycle, Cancer”, which contains approximately 60% of the identified differentially expressed proteins (Figure S3B). Notably, the oncogene c-myc, which was one of the hubs in the built network (Figure S3B), is overexpressed in DU145R80 compared to DU145 cells and correlates with the acquisition of an invasive phenotype [8]. Cancer was confirmed as the IPA-predicted top altered disorder, while the IPA-predicted top biological functions of the identified proteins were “Cell morphology”, “Cell-To-Cell Signaling and Interaction”, “Cellular Assembly and Organization and Cellular Movement” (Table 2).

**Table 2:** Ingenuity pathway analysis-predicted top biological functions

Top Biological Functions	*p* Value (ranging from)^a^	No. of molecules^b^
**Disease and Disorders**		
Cancer	7,30 E-06 – 3,84 E-02	8
Gastrointestinal Disease	7,30 E-06 – 1,95 E-03	5
Hepatic System Disease	7,30 E-06 –7,30 E-06	3
Connective Tissue Disorders	1,87 E-05 – 2,32 E-02	8
Hereditary Disorders	1,87 E-05 – 1,90 E-02	7
**Molecular and Cellular Functions**		
Cell Morphology	9,77 E-04 – 3,77 E-02	3
Cell-To-Cell Signaling ad Interaction	9,77 E-04 – 4,87 E-02	2
Cellular Assembly and Organization	9,77 E-04 – 3,77 E-02	4
Cellular Function and Maintenance	9,77 E-04 – 3,77 E-02	3
Cellular Movement	9,77 E-04 – 1,26 E-02	3
**Physiological System Development and Function**		
Hematological System Development and Function	9,77 E-04 – 4,87 E-02	1
Immune Cell Trafficking	9,77 E-04 – 4,87 E-02	1
Tissue Development	9,77 E-04 – 4,87 E-02	2
Organismal Survival	1,31 E-03 – 1,31 E-03	3
Cardiovascular System Development	1,95 E-03– 2,32 E-02	2

a Fisher's exact test was used to calculate a *p* value for each protein of the data set identified in the biological function studied, indicating the probability that each biological function assigned to the data set is assigned by chance; then we have a range of *p* values corresponding to all *p* values calculated for all proteins at the dataset in the biological function.

b The number of molecules of the 15 differentially expressed protein dataset is shown.

**Figure 3:**
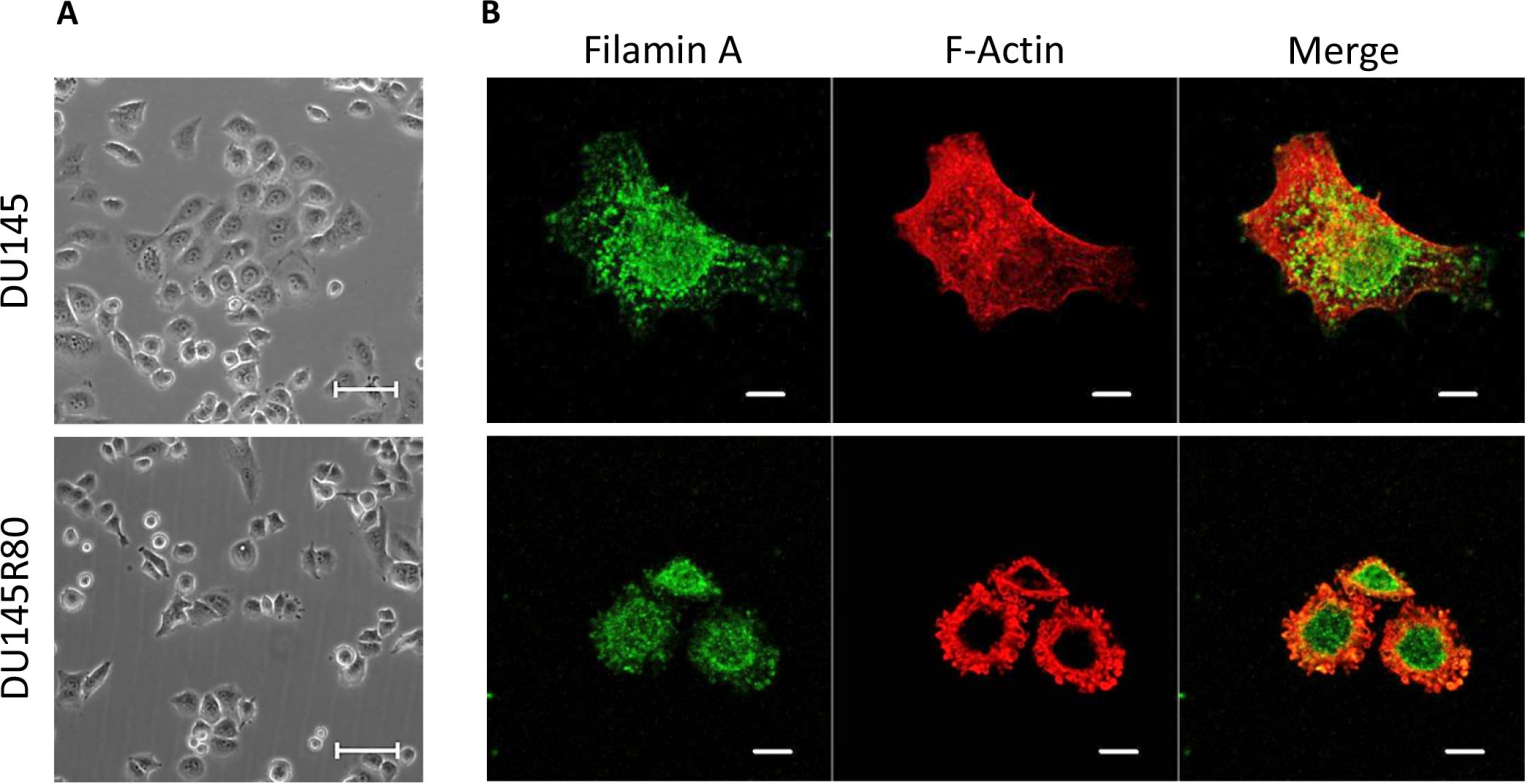
Morphology of DU145 and DU145R80 cells and cellular localization of FLMNA and F-actin **(A)** Representative images of DU145 and DU14580 cells grown on glass slides to semi-confluence and then analysed by phase contrast microscopy; or **(B)** by confocal microscopy after double staining for FLMNA and F-actin. Original magnifications: 200x (A) or 630x (B). Scale bars: 50 μm (A) or 10 μm (B).

**Figure 4:**
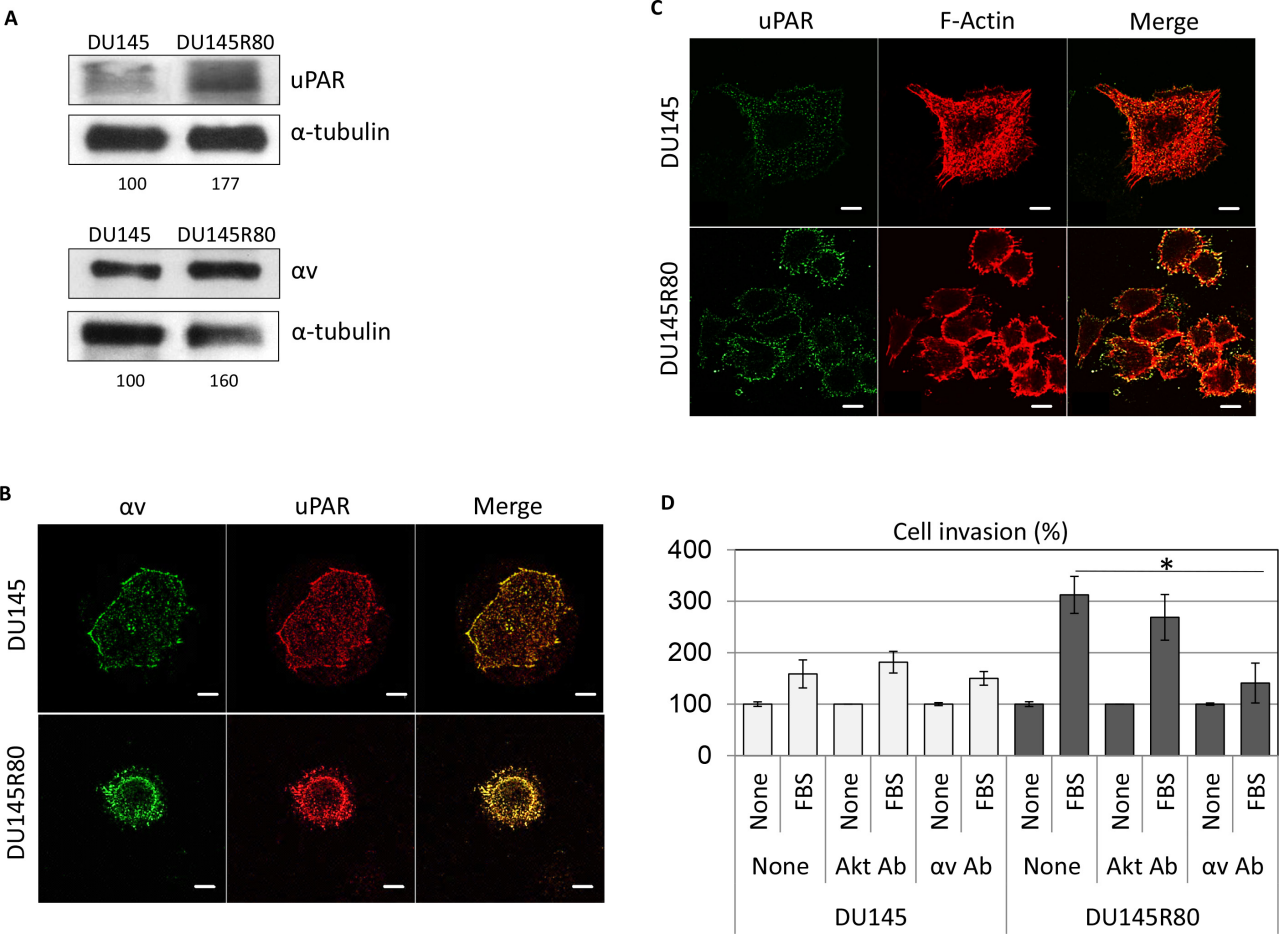
Involvement of αv integrin in cell invasion ability of DU145R80 cells expressing higher levels of uPAR and αv as compared to DU145 cells **(A)** Western blot analysis of uPAR and αv integrin proteins evaluated after 48 h of cell culture; **(B)** representative images of DU145 and DU14580 cells grown on glass slides and analyzed by confocal microscopy after double staining for αv and uPAR; or **(C)** uPAR and F-actin. Original magnifications: 630x. Scale bars:10 μm. **(D)** Cell invasion of DU145 and DU145R80 cells toward 10% FBS, with/without 1:500 anti-αv or anti-Akt monoclonal antibodies, the last used as antibody negative control. The extent of invasion is expressed as a percentage of the basal cell invasion assessed in the absence of chemo-attractants, considered as 100% (None). Data represent the mean ± SD of three experiments in duplicate. *Statistical significance calculated against the positive control with *p* = 0.0003.

The molecular and cellular functions of the identified proteins prompted us to characterize the morphology of DU145R80 compared to DU145 cells. As shown in Figure 3A, DU145 and DU145R80 cell lines show a completely different morphology. DU145 cells exhibit an epithelial, flattened morphology with a circumferential margin closely adherent along their lateral and apical surfaces. In contrast, DU145R80 cells assume a smaller, rounded morphology and reduced cell-cell contact, in agreement with the epithelial-to-mesenchymal transition (EMT) feature of this cell line [8]. These changes in cell morphology reflect the reorganization of the F-actin cytoskeleton of DU145R80 cells with the appearance of condensed actin-rich structures, consisting of membrane-bound ‘ring’ domains localized at the cell periphery resembling podosomes/invadopodia (Figure 3B). Among cytoskeleton regulating proteins, FLNA is a major actin crosslinking protein and signaling scaffold with important roles in regulating cell migration. Thus, we analyzed the intracellular distributions of FLNA in DU145 and DU145R80 cell lines. As shown in Figure 3B, FLNA appears dispersed in the cytoplasm of DU145 cells, apparently more concentrated in the nuclei and rarely co-localized with actin filaments. Conversely, FLNA is less dispersed in the cytoplasm of DU145R80 cells and clearly co-localizes with actin-rich structures resembling podosomes and/or invadopodia (Figure 3B). FLNA links the peripheral actin cytoskeleton to integrins [9], which represent a critical functional link between cytoskeleton reorganization and the extracellular matrix. Recent studies have shown that αv-containing integrins are functionally involved in the maintenance of a highly migratory and mesenchymal cellular phenotype in human PCa cells. Furthermore, we and others have previously documented that: i) αv-integrin activity is tightly modulated by uPAR which promotes cell migration by interacting with the α chain of αv-integrins [10]; ii) uPAR overexpression in human PCa is associated with metastatic disease and poor prognosis [11]. Accordingly with their pivotal role in determining the acquisition of an invasive phenotype, DU145R80 cells exhibit a clear-cut increase in both αv-integrin and uPAR expression levels compared to DU145 cells (Figure 4A).

Interestingly, the actin-rich structures localized at the cell periphery of DU145R80 cells are rich in uPAR (Figure 4C), which co-localizes with αv-integrin (Figure 4B). According to the immunoblotting data, DU145 cells express low levels of uPAR and αv-integrin, mostly localized along the entire cell surface (Figure 4A and 4B).

Furthermore, in keeping with the previously reported higher invasive ability of DU145R80 cells [8], we found that DU145 and DU145R80 cells exhibit a low and high ability to invade matrigel in the presence of FBS, (159% and 312% of the basal cell invasion, respectively) (Figure 4D). Interestingly, when anti-αv antibody was added to the cell suspension, the capability of DU145 and DU145R80 cells to cross matrigel became comparable, indicating that αv-integrin is deeply involved in the acquisition of an invasive phenotype by DU145R80 cells. As shown in Figure 4D, cell exposure to anti-αv antibodies causes 55% reduction of FBS-dependent DU145R80 cell invasion while DU145 cell invasiveness was unchanged.

**Figure 5:**
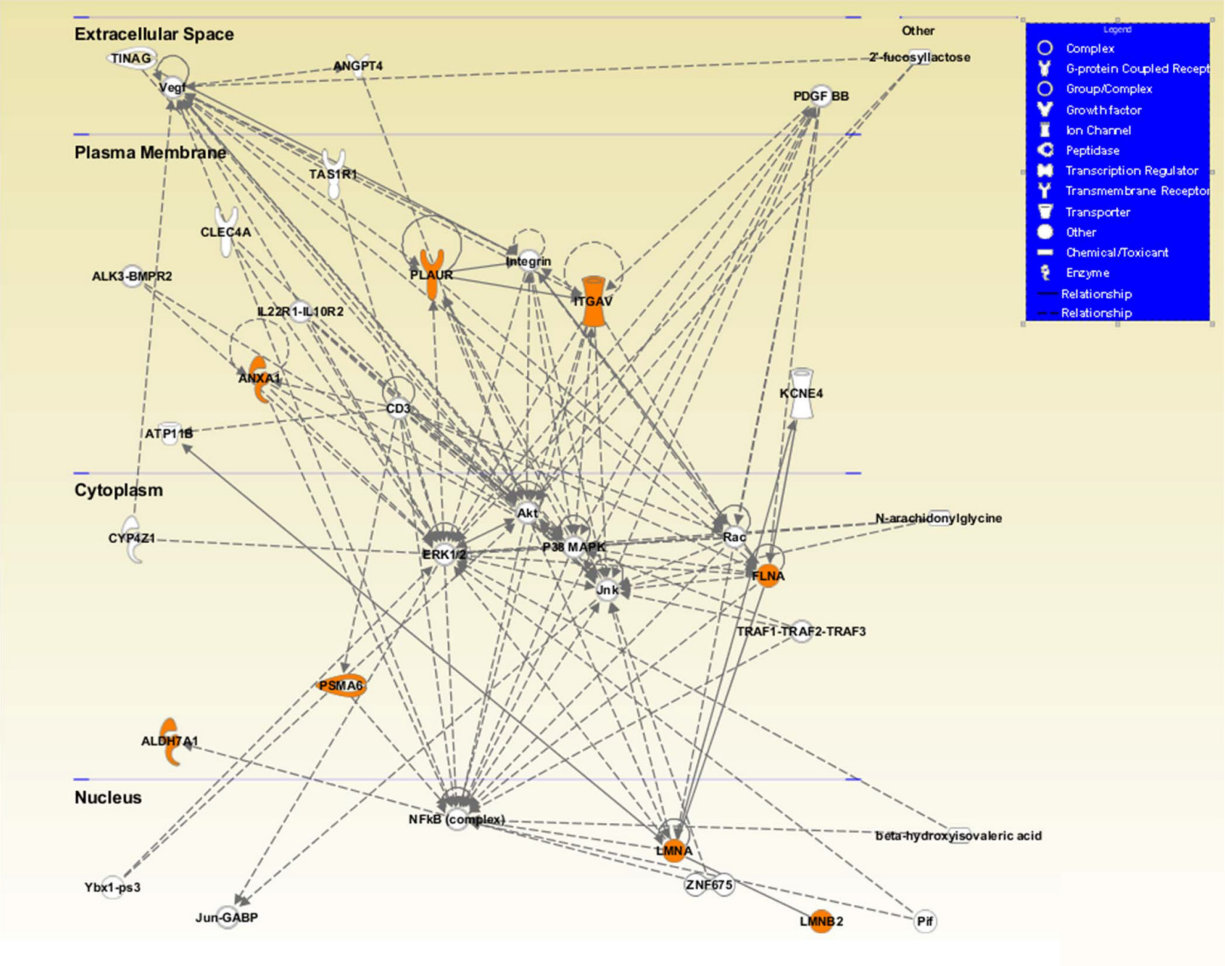
Visual representation of the principal network generated by Ingenuity Pathway Analysis (IPA) The network included 6 of the 7 validated proteins with direct and indirect relations (ANXA1, FLMNA, PSMA6, ALDH7A1, LMNA, LMNB2) connected with both αv (ITGAV) and uPAR (PLAUR) proteins and localized to a specific cell compartment (all colored in orange). Network proteins are visualized by proper symbols, which specify the functional nature of protein. Each node represents a protein and its direct (represented by solid lines) and indirect (represented by dotted lines) association with other proteins. Proteins with no background color were undetected in the study but have been inserted by IPA to produce a highly connected network.

Finally, we performed an additional IPA analysis showing a cellular network that included 6 of the 7 validated proteins connected with both αv-integrin and uPAR, and localized to a specific cell compartment (Figure 5).

All together, these findings strongly support the notion that, unlike DU145 cells, DU145R80 cells, as a consequence of cytoskeleton re-organization, display actin-rich structures localized at the cell periphery where αv-integrin activation triggers the acquisition of an invasive phenotype.

### Oncomine analysis demonstrated close correlation between our experimental data and publically available tumor expression profiling data from PCa patients

To evaluate the eventual clinical impact of our results, we performed data mining and analyzed the gene expression of all the up-regulated and validated proteins by using the publicly available Oncomine database (http://www.oncomine.org) [12].

We required a *p*-value of ≤ 0.05 and a change of at least 1.3 folds, to interrogate 6 human prostate cancer datasets for tumor mRNA expression compared to adjacent non-tumor (or normal) tissues. Interestingly, as shown in Table 3, the results indicated that a significant increase in mRNA levels, expressed as fold change of cancer vs normal, was observed for all the up-regulated and validated proteins (ANXA1, ALDH7A1, LMNA, LMNB2, eEF1γ and uPAR) and correlated also quite well with the fold change of DU145R80 vs DU145 presented in Table 1 and reported also in Table 3. Similarly, significantly lower mRNA expression of PMSA6 and FLNA, both down-regulated in DU145R80 cells, was detected in prostate tumor tissue *vs* normal tissue. PSMA6 and FLMNA down-regulation also correlates well with poor Gleason score (Figure S4 A-B). Of note, ANXA1 and uPAR as well as LMNA and eEF1γ mRNA levels are upregulated in the same datasets. Taken together, these data indicated that the modulated proteins could participate in carcinogenesis of prostate cancer and suggest that the expression of at least some of them might correlate with prognosis.

**Table 3:** Oncomine data

Gene	Tumor type	Fold change cancer vs normal	Fold change DU145R80 vs DU145	Patients number	*P* value	Study (ref.)
FLNA	Prostate carcinoma	−1.923	−1.51	185	8.61E-11	[13]
ANXA1	Prostate carcinoma	1.460	1.54	19	0.009	[14]
PSMA6	Prostate adenocarcinoma	−1.610	−1.4	89	7.16E-5	[15]
ALDH7A1	Prostate adenocarcinoma	1.355	2.01	40	0.003	[16]
LMNB2	Prostate carcinoma	1.880	2.32	15	0.046	[17]
LMNA	Prostate carcinoma	1.825	1.70	102	0.016	[18]
eEF1g	Prostate carcinoma	2.978	1.54	102	5.30E-5	[18]
UPAR/PLAUR	Prostate carcinoma	2.025	1.77*	19	0.017	[14]

Data summarized in the table was obtained from cDNA microarray analysis published in Oncomine databases. In particular for each gene was reported the name, the prostate tumor type, the fold change, the *P* value and number of patients referred to the study analyzed.

* For UPAR/PLAUR the fold change was calculated based on immunoblot quantization (DU145R80/DU145 expression level).

## DISCUSSION

PCa, particularly in the castration-resistant stage, is a tumor difficult to treat for which no definitive molecular markers of prognosis or predictive of tumor response to treatment have yet been identified.

In this study, we have performed a quantitative proteomic analysis using 2-DE DIGE coupled with MS as an attempt to better characterize an isogenic model of DU145 PCa cells and their derived ZOL-resistant, aggressive subline DU145R80, previously selected by our group [8]. The unsupervised multivariate analyses using PCA indicated a high reproducibility between sample replicates and demonstrated distinct expression patterns from the two experimental groups. In addition, the 2-DE DIGE results are based on very stringent statistical parameters and indeed by IPA analysis we demonstrated a strong correlation among the MS identified proteins (up 80%) consistently corroborating the biological reliability of our results. We identified 15 proteins differentially expressed between the two cell lines, 7 of which were also validated by Western blotting. The molecular and cellular functions of the identified proteins were homogeneous and prevalently related with regulation of cell morphology, organization, movement or cell-to-cell interaction. Indeed, although DU145R80 was derived from DU145 upon the sole selection in ZOL [8] and the number of differentially expressed proteins is small, phenotypic analysis confirmed a strikingly different morphology between the two cell lines. The smaller, rounded and reduced cell-cell contact morphology of DU145R80 cells, most likely reflecting the reorganization of the F-actin cytoskeleton, is consistent with the identified differentially expressed proteins by this cell line compared with the parental DU145 line and is well correlated with their increased invasive capability and EMT features we have described before [8]. Notably, the modulation of the identified proteins also, correlated with increased tumor expression and tumor aggressiveness in publicly available prostate cancer genomic data.

In detail, the structural proteins (34% of the total proteins identified) differentially expressed between the two cell lines could be divided into proteins involved in cytoskeleton organization (20%) and components of the nuclear lamina (14%).

Among cytoskeleton regulating proteins down-regulated in DU145R80 compared with DU145 cells, FLNA couples cell cytoskeleton to extracellular matrix and integrin receptor signaling by crosslinking actin filaments and is considered a critical protein in cell adhesion, spreading, and migration [9]. In addition, FLNA acts as a scaffold for intracellular proteins involved in signal transduction, interacting with more than 45 proteins with diverse functions [9]. The function of FLNA in carcinogenesis and/or in cancer cell migration and invasion is not fully understood. Interestingly, it has been shown that FLNA down-regulation promotes matrix metalloproteinase secretion [19], a mechanism that may contribute to the association of FLNA down-regulation and cancer. In this regard, we have previously reported that the increased invasive capability of DU145R80 cells was paralleled by the increase in MMP9 gene expression and secretion in culture media [8], and we confirmed DU145R80 cells exhibit higher FBS-dependent invasive ability in matrigel compared to DU145 cells.

Conversely, another study in PCa suggested that the localization rather than the expression of FLNA is crucial and that a substantial increase in cytoplasmic staining for FLNA in PCa metastatic tissue suggests a role for FLNA in PCa aggressiveness, likely involving cell migration [20]. An unanticipated role of cytoskeletal proteins such as FLNA in the nucleus has also been suggested by the findings that FLNA interacts with androgen receptor (AR) and suppresses AR transcriptional activity [20] and by a recent report showing that FLNA plays a role in the repair of a variety types of DNA damage and that lack of FLNA increases cell sensitivity to DNA damage-based cancer therapy [21]. Although from our confocal microscopy experiment, we cannot perform a quantitative evaluation of FLNA distribution between nucleus and cytoplasm, it is evident that in DU145 cells, FLNA aggregates are apparently more concentrated in the nuclei, dispersed in the cytoplasm and rarely co-localized with actin filaments, while in DU145R80 cells FLNA appears to be mostly localized in the numerous podosomes and/or invadopodia. Invadopodia are ventral membrane protrusions through which invasive cancer cells degrade the extracellular matrix and it was shown that invadopodia are the sites where lipid raft formation and trafficking occur [22]. Notably EMT induces a massive rearrangements of the actin cytoskeleton as well as changes in cell-cell and cell-ECM junctions and recently it was suggested that EMT could provoke an assembly of podosomes and invadopodia [23]. Furthermore, a proteomic study on prostate needle biopsy specimens also found FLNA markedly downregulated in PCa *vs* benign prostatic hyperplasia (BPH) [24], whereas a gene expression profiling study found low levels of endogenous FLNA in advanced and aggressive PCa compared with benign tissues [13] (Table 3 and Figure S4).

Several transmembrane and signaling molecules have been reported to interact with FLNA including integrins [9, 25]. In this regard, we found higher expression levels of αv-integrin in DU145R80 compared with DU145 cells, suggesting that it has a role in the different phenotypes displayed by the two cell lines. We have previously shown that uPAR/αvβ5 vitronectin receptor interaction positively regulates tumor cell migration and invasion in breast cancer cells [26]. In PCa cells, uPAR interacts and activates αvβ3 integrin, leading to cytoskeletal reorganization and increased cell motility [27]. Thus, uPAR not only focuses urokinase-dependent proteolytic activity at leading edge of invading cells but also signals to cytoskeleton through formation of complex multiprotein units containing integrins. Indeed we also demonstrated the overexpression of uPAR, in DU145R80 compared to DU145. Notably, uPAR overexpression has been also confirmed in PCa tissues [14] (Table 3). Significantly, uPAR mostly co-localized with actin fibers at the cell surface in DU145R80 cells. We could speculate that in DU145R80 invadopodia-like structures, uPAR ultimately impact on αv-integrin activation resulting in the acquisition of a more aggressive phenotype. This hypothesis is also suggested by our data demonstrating that DU145 and DU145R80 cell ability to cross matrigel became comparable upon exposure to blocking αv antibodies. However, DU145 cell invasion was not affected by anti-αv antibodies raising the possibility that other signaling components may be involved. It could be the case of the metalloproteases MMP-2 that are overexpressed by DU145R80 compared to DU145 cells [8] and have been shown to bind integrin αvβ3 [28].

Another critical structural protein we found up-regulated in DU145R80 is ANXA1, a calcium-binding (‘annexing’) anti-inflammatory protein that acts extracellularly as a strong inhibitor of eicosanoid synthesis and phospholipase A2 and that has intracellular roles in many diverse functions, such as membrane and vesicle trafficking, exocytosis, phagocytosis, signal transduction, proliferation, differentiation and apoptosis [29]. Altered expression profiles, phosphorylation, subcellular localization, and/or specific modulation of mitogenic signals are all possible mechanisms by which ANXA1 protein could mediate its biological effects. However, the exact mechanisms through which ANXA1 exerts some or all of its effects, particularly in cancer, are still not clearly understood. Indeed, several findings concerning its role in tumorigenesis are controversial because its expression is reduced in certain cancers, while it is increased in others [30]. However, ANXA1 overexpression is associated with metastasis and poor prognosis in multiple malignancies, including prostate cancers [14, 31, 32] (Table 3). Furthermore, there is a growing body of evidence indicating that ANXA1 may interact with cytoskeletal proteins such as tubulin and actin and that these interactions could be implicated in the mechanism of migration and invasion [33, 34]. Interestingly, it was shown that ANXA1 is a substrate of the EGFR tyrosine kinase, that ANXA1 is required for EGFR trafficking and that EGF-induced phosphorylation of ANXA1 triggers its co-localization with F-actin in the lamellipodia [35]. We have recently found that ANXA1 and F-actin co-localize in DU145R80 but not in DU145 cells (Petrella A, Pucci B, Milone M. R. and Budillon A, unpublished data). Moreover, it was shown that the regulatory action of cell surface or extracellular ANXA1 is mediated by signaling through formyl peptide receptors (FPRs) [36–38]. Notably, FPRs form complete signaling units with extracellular matrix proteins such as vitronectin and with membrane receptors such as uPAR and integrins, which stimulate actin filament assembly and reorganization that are critical events underlying cell migration [39–41].

Finally, a novel function of ANXA1 as a potential regulator of pathological angiogenesis was recently unveiled by demonstrating that ANXA1 is a new target of the p38-MAPK pathway and that ANXA1 regulates endothelial cell migration in response to vascular endothelial growth factor (VEGF) [42]. In this regard, we have previously reported that activation of p38-MAPK pathway has a critical role in the induction of ZOL resistance, as well as in the acquisition of a more aggressive and invasive phenotype demonstrated in DU145R80 cells. Notably in the IPA network showed in Figure 5, p38-MAPK is one of the main hubs.

We also found increased expression of two nuclear lamin proteins, LMNA and LMNB2, in DU145R80 compared to DU145 cells. LMNA and LMNB2 belong to the lamins, the major architectural proteins of the cell nucleus, which are essential for determining global nuclear organization, regulating the movement of macromolecules into and out of the nucleus and also providing anchoring sites for chromatin domains, particularly those that are enriched in silenced genes. More recently, a growing number of human diseases have been recognized that are linked to defects in nuclear envelope-specific proteins. Notably, some of these disorders revealed that the nuclear envelope not only provides a foundation that defines nuclear architecture as a whole but also has significant roles in cytoplasmic organization and cytoskeletal mechanics [43].

Although lamin proteins are often aberrantly expressed or localized in tumors, the nature of lamin function in cancer is still unclear. It was shown that LMNA proteins are positively involved in the malignant behavior of PCa; indeed the overexpression of LMNA resulted in stimulation of cell growth, colony formation, migration and invasion in the PCa cell lines LNCaP, DU145, and PC3 [44]. Overexpression of both LMNA and LMNB2 was reported in PCa tissues [17, 18] (Table 3). Moreover, the presence of lamin isoform A is related to increased invasiveness and EMT by indirect down-regulation of E-cadherin [45].

Among other proteins differentially expressed between DU145R80 and DU145 cells, 6% were involved in protein fate, 40% were involved in RNA processing and 20% were involved in the regulation of metabolism. Some of these proteins were described as aberrantly expressed in cancer cells, associated with tumorigenesis and/or with bad prognosis in several cancer types including PCa [15, 16, 18] (Table 3). Among the validated proteins, PSMA6, down-regulated in DUR145R80 compared with DU145, is a very large 2.4 KDa ATP-dependent proteolytic complex that is found in the cytoplasm and nucleus of eukaryotic cells. Of note many proteasome substrates are known mediators of pathways that are dysregulated with neoplasia [46]. The eEF1γ, upregulated in DU145R80, physically interacts with the RNA polymerase II (pol II) core subunit 3 (RPB3), both in isolation and in the context of the holoenzyme. Importantly, eEF1γ has recently been shown to bind vimentin promoter, and depletion of eEF1γ causes the vimentin protein to be incorrectly compartmentalized and to severely compromise cellular shape and mitochondria localization [47]. According to these observations, eEF1γ overexpression might, at least in part, explain the up-regulation of vimentin we have previous observed in DU145R80 [8]. ALDH7A1, up-regulated in DU145R80, is known as an enzyme involved in the detoxification of aldehydes generated by alcohol metabolism and lipid peroxidation, but has been also recently shown to be functionally involved in PCa bone metastasis [48]. Interestingly, the altered expression of both PSMA6 and of eEF1γ in DU14R80 can in turn affect post-trascriptionally (regulating protein degradation or protein translation, respectively) the expression of the other proteins, thus explaining some discrepancy between mRNA and protein expression (Figure S2).

In conclusion, we unveil a signaling network that physically links the interior of the nucleus via the cytoskeleton to the extracellular matrix and that could link changes in the cytoskeleton dynamics with altered protein expression and dictate PCa aggressiveness. This network is highlighted by the IPA analysis reported in Figure 5 that included 6 of the 7 validated proteins connected with both αv and uPAR, and localized to a specific cell compartment.

This signaling network is therapeutically relevant for cancer but potentially also for other disorders. Indeed, although cancer was confirmed as the IPA-predicted top altered disorder, also connective tissue and hereditary disorders involved a large number of the identified proteins (Table 3). For example loss of function of FLNA has been associated with a wide spectrum of connective tissue and vascular abnormalities [49], glucocorticoid-induced ANXA1 induction has been recently related with immune-mediated inflammation and the pathogenesis of reumathoid artritis [50], laminA recently has been correlated with immune cell functions [51], while both A and B type lamin mutations and/or defects in their expression or post-translational processing, cause a heterogeneous group of diseases known as laminopathies [52].

Overall, on the basis of our findings, we highlighted novel candidate markers that could identify aggressive PCa phenotypes, that be used to monitor anticancer therapy in PCa patients, and/or that represent novel targets for advanced PCa stage therapy and for preventing/overcoming the acquisition of resistance to ZOL treatment.

## MATERIAL AND METHODS

### Cell lines

Cell line DU145, derived from prostate cell line models, was purchased from American Type Culture Collections (Rockville, MD, USA). ZOL-resistant DU145R80 cells were obtained as previously described [8]. Both cell lines were cultured as previously described [8].

### Protein preparation and labeling with DIGE dyes

All 2-DE DIGE reagents and instruments were provided by GE Healthcare Bio-Sciences, Pittsburgh, USA. Proteomic experiments were performed as described by Bianchi et al. [53] with some modifications. The experimental methodology used for 2-DE DIGE analysis and protein identification is shown in Supplementary Figure S1. Briefly, a biological quadruplicate (four independent biological conditions) were used to give statistic confidence to 2-DE DIGE data. DU145 (1 × 10^6^ cells) and DU145R80 (1.5 × 10^6^ cells) were seeded on 100-mm tissue culture plastic dishes. Cells were collected using 1X PBS, washed twice with PBS containing 250 mM sucrose and stored at −80°C until use. Each sample was lysed in 8 M urea, 4% chaps and 40 mM DTT. After 3 h of shaking at RT, the samples were sonicated with ultrasonic waves (amplitude 80 %, pulse 20 for 6 sec three times) on ice. Protein concentration was evaluated by Bradford method assay [54]. Bovine serum albumin (BSA) was used as the standard for the calibration curve. Proteins were precipitated using the 2-D Clean-Up Kit (GE Healthcare) to remove additional salts according to the manufacturer's instructions. Minimal protein labeling for 2-DE DIGE was performed according to the manufacturer's instructions (CyDye DIGE fluor minimal dye). Briefly, after precipitation, proteins from DU145 and DU145R80 were resuspended in 7 M urea, 2 M thiourea, 4% w/v chaps and 25 mM Tris and then buffered to between 8–9 pH. Protein concentration was estimated by the Bradford assay as described before. After quantification, the protein samples were labeled using the fluorescent CyDyes™ (Cy2, Cy3, and Cy5) developed for DIGE (GE Healthcare). Gel-to-gel variation was controlled by using an internal standard (IS) sample obtained by mixing equal amounts of proteins from all the analyzed samples and labeled with Cy2 minimal dye. The experimental design using the three-dye approach is illustrated in Supplementary Table S1. Briefly, 50 μg of IS containing an equal amount of sample proteins was labeled with 400 pmol Cy2, and the two 50 μg protein samples were labeled with 400 pmol Cy3 or Cy5. Dye-swapping among protein samples was conducted to avoid artifacts due to preferential labeling. Protein samples were kept on ice and fluorescently labeled in the dark for 30 min. The reaction was then quenched by the addition of 1 μl 10 mM l-lysine (GE Healthcare) and the samples were kept on ice in the dark for 10min.

### 2-DE DIGE

Isoelectrofocusing (IEF) was carried out on preformed immobilized non-linear pH 3–10 gradient gels of 24 cm length using an Ettan IPGphor system. In detail, Cy2-, Cy3-, or Cy5-labeled proteins (50 μg each) were pooled and added to the rehydration buffer to obtain a final volume of 450 μL (8 M urea, 4% chaps, 1% Pharmalytes, 1% DTT, 0.01% bromophenol blue). The buffer was then added to the strip and left for 16 h at RT to ensure a complete rehydration. The IEF run was performed according to the following electrical conditions at 20°C and 50 μA/strip in the dark. Step 1: step and hold 100 V for 4 h; step 2: gradient 1000 V for 6 h; step 3: gradient 8000 V for 3 h; step 4: step and hold 8000 V for 4.40 h. After IEF, 1^st^ dimension strips were subjected to two equilibration steps: 15 min in 6 M urea, 2% w/v SDS, 2% w/v DTT, 3% v/v glycerol, and 0.05 MTris-HCL pH 6.8 followed by 15 min in 6 M urea, 2% w/v SDS, 2.5% w/v iodoacetamide, 30% v/v glycerol, 0.05 M Tris-HCL pH 6.8, and 0.01% bromophenol blue. The 2^nd^ dimension was then performed on 12% SDS-PAGE homogenous polyacrylamide gels (24 cm × 20 cm × 1 mm) using the EttanDalt Twelve Separation Unit. Gels were cast between low fluorescent Pyrex glass plates (GE Healthcare) to minimize background fluorescence during scanning. Electrophoresis was performed as follows. Gels were run in the dark at 23°C according to the following power steps: 2 W/gel for 30 min followed by 17 W/gel for 4 h, or until the dye front reached the bottom of the gel.

### 2-DE DIGE image acquisition, analysis, and processing

Fluorescence signals were imaged by Typhoon Trio laser densitometer recording band-pass filtered emission wavelengths of 520 nm (Cy2), 580 nm (Cy3), and 670 nm (Cy5). Gels were scanned at 100 μm resolution and the photomultiplier tube voltage was set to values ranging between 500 and 700 V to ensure pixel intensity maxima between 50,000 and 80,000 pixels for the three dyes. Spot identification, background elimination, point matching, and differential analysis of the protein spots were completed using a 2D DeCyder software (Differential In-Gel Analysis and Biological Variance Analysis software module; V7.1, GE Healthcare). Each gel image was processed in the Differential In-gel Analysis (DIA) module of DeCyder prior to export to the Biological Variation Analysis (BVA). In DIA, spot detection was performed based on an estimated 10,000 spots. Background subtraction and in-gel normalization processes were carried out automatically by the software. The DIA workspaces were imported into BVA for manual spot matching to the master gel. This BVA was also employed to calculate mean average ratios and statistical analysis between groups. The Student's t-test was used to calculate significant differences in the relative abundance of individual protein spot features between two groups in the 2-DE DIGE analysis. All missing values were treated as absent rather than given a value of zero. Only the spots found to be statistically significant ( *p* ≤ 0.05) and ratio ≥ or ≤ 1.4 were isolated for further investigation. These two processes were performed automatically using the Batch Processor Module, and the final result is a list that contains the proteins differentially expressed between the two groups analyzed. These proteins will be picked from the preparative gels as described below. Multivariate analysis was performed to assess global changes in DU145R80 compared to DU145. The DeCyder EDA (Extended Data Analysis, V7.1) module was used for the principal component analysis (PCA) that eliminates redundant variables and reduces data complexity.

### Protein identification

Protein extracts were separated onto preparative gels, and then proteins of interest were recovered from the gels for identification. Proteins (about 500 μg) from four sample groups were resolved on separate preparative polyacrylamide gels and were visualized by staining with Deep Purple Total Protein Stain according to the manufacturer's instruction (GE Healthcare). In detail, the gels were fixed with a solution containing 7.5 % acetic acid/10 % methanol, at RT with gentle agitation overnight. The next day, the fixation solution was washed out and replaced with washing solution (35 mM NaHCO3, 300 mM Na2CO3 in water) and shaken for 30 min at RT. After removal of the washing solution, the gels were stained with the Deep Purple solution (1:200 dilution in distilled water) for 1 h at RT with gentle agitation in the dark. The stain solution was then replaced with 7.5 % acetic acid and incubated at RT for 15 min (wash solution). The gels were imaged at this stage by setting the Typhoon scanner as described: excitation Green laser (532 nm) and emission 560 LP or 610 BP filter. Once the preparative gels were imaged, a match process was made between these images and spot maps previously analyzed by DeCyder 2D 7.1 software, with the aim being to find the protein spots included in the list. In this way, the X/Y coordinates of the spots given by DeCyder were acquired and used by an Ettan Spot Picker robot instrument to pick spots from preparative gels and then identified the spots by mass spectrometry. Selected protein spots stained by Deep Purple and excised from the gel were washed in 50 mM ammonium bicarbonate pH 8.0 in 50% acetonitrile to a complete destaining. The gel pieces were re-suspended in 50 mM ammonium bicarbonate pH 8.0 containing 100 ng of trypsin and incubated for 2 h at 4°C and overnight at 37°C. The supernatant containing the resulting peptide mixtures was removed and the gel pieces were re-extracted with acetonitrile. The two fractions were then collected and freeze-dried. The peptide mixtures were analyzed by LC-MS/MS using the LC/MSD Trap XCT Ultra (Agilent Technologies, Palo Alto, CA) equipped with a 1100 HPLC system and a chip cube (Agilent Technologies). After loading, the peptide mixture (7 μl in 0.2% HCOOH) was first concentrated at 4 μl/min in a 40 nl enrichment column (Agilent Technologies chip), with 0.1% formic acid as the eluent. The sample was then fractionated on a C18 reverse-phase capillary column (75 μm × 43 mm in the Agilent Technologies chip) at a flow rate of 300 μl/min, with a linear gradient of eluent B (0.1% formic acid in acetonitrile) in A (0.1% formic acid) from 7 to 50% in 35 min. Elution was monitored on the mass spectrometers without any splitting device. Peptide analysis was performed using data-dependent acquisition of one MS scan (m/z range from 400 to 2000) followed by MS/MS scans of the three most abundant ions in each MS scan. Dynamic exclusion was used to acquire a more complete survey of the peptides by automatic recognition and temporary exclusion (2 min) of ions from which definitive mass spectral data had previously been acquired. Moreover, a permanent exclusion list of the most frequent peptide contaminants (keratins and trypsin peptides) was included in the acquisition method to focus the analyses of significant data. Mass spectral data obtained from the LC-MS/MS analyses were used to search a non-redundant protein database using an in-house version of the MASCOT (Matrix Science, Boston, MA, USA) software. Peptide mass values and sequence information from LC-MS/MS experiments were used in the MS/MS ion search taking into account the carbamidomethyl-Cys as a fixed modification, oxidation of methionine as a possible modification, a number of missed cleavages of 1 and precursor ion and fragment ion mass tolerances of ± 600 ppm and 0.6 Da, respectively.

### Immunoblotting

To validate the results of the proteomic studies, the protein samples for the 2-DE DIGE study were examined by 1-D or 2-D immunoblotting. In brief, 60 μg protein samples were separated by 1-D SDS-PAGE and transferred to a nitrocellulose membranes. For 2-D immunoblotting, IEF was performed as described for 2-DE DIGE with some differences. Protein samples (100 μg) were loaded on 7 cm length preformed immobilized non-linear pH 3–10 gradient strips passively rehydrated using 125 μL rehydration buffer as described before. IEF was performed according to the following electrical conditions at 20°C and (50 uA/strip). Step1: step and hold 300 V for 0.01 h; step2: gradient 1000 V for 0.30 h; step3: gradient 5000 V for 1.30 h; step4: step and hold 5000 V for 3.06 h. For the second dimension, strips were loaded on mini-gel Bio-Rad systems (Hercules, California, USA) and electrophoretically transferred to a 0.45 μm nitrocellulose membrane (GE Healthcare) in the Bio-Rad ElectroBlot system as described for 1-D SDS-PAGE. Primary antibodies were purchased as follows: Cdk4 (#sc-260, 1:1000), from Santa Cruz Biotechnology, Inc. San Jose, CA, USA); ALDH7A1 (#ab53278, 1:5000) and eEF1γ (#ab124994, 1:1000) were from Abcam, Cambridge, UK; Filamin A (#4762, 1:1000), Lamin B2 (#9622, 1:1000), Lamin A/C (#2032, 1:1000), PSMA6 (#2459, 1:1000), Annexin-A1 (#3299, 1:1000), and α-tubulin (#2144, 1:1000) were from CST, Danvers, MA, USA. αv-integrin (#MAB1960, 1:1000) was from Chemicon International, Inc. Temecula, CA. An anti-uPAR monoclonal antibody was kindly provided by G. Hoyer-Hansen (Finsen Institute, Copenhagen, Denmark).

Secondary antibodies were purchased as follows: polyclonal swine anti-rabbit immunoglobulins/horseradish peroxidase (HRP)–linked IgG secondary antibody conjugate and polyclonal rabbit anti-goat immunoglobulins/HRP conjugate were from Dako-Cytomation (#P0217, 1:2000, Glostrup. Denmark), and rabbit polyclonal anti-mouse IgG H&L HRP conjugate (#ab6728, 1:2000) was from Abcam. Enhanced chemiluminescence (ECL) immunodetection reagents were from GE Healthcare. The chemiluminescent signal was detected with Image Quant LAS 500, and the intensity was measured by ImageQuantTL image software (GE Healthcare).

### RNA isolation and quantitative RT-PCR assay

Real-time PCR (RT-PCR) was performed as described by Milone et al. [8]. Briefly, Total RNA was isolated using the RNeasy plus mini kit (Qiagen, Hilden, Germany) as indicated by the manufacturer's instructions and quantified using a NanoVue Plus spectrophotometer (GE Healthcare). Reverse transcription was performed using the QuantiTect Reverse Transcription Kit (Qiagen). Taqman probes were used to quantify RNA levels specific for GUSB (GUSB_Hs00939627_m1), ALDH7A1 (Hs00609622_m1), eEF1gamma (eEf1gamma_Hs01922638_v1), PSMA6 (PSMA6_Hs00853240_sH), LMNB2 (LMNB2_Hs00383326_m1), LMNA (LMNA_Hs00153462_m1), ANXA1 (ANXA1_Hs00167549_m1), and FLNA (FLNA_Hs00924645_m1) (Applied Biosystems, Foster City, CA, USA). Cycle threshold values (Ct) generated using Sequence Detection System 2.2.2 (Applied Biosystems, Foster City, CA, USA) default parameters were exported to determine relative mRNA abundances among genes in the classifier. All gene expression levels were normalized to GUSB expression. Each sample was tested in triplicate using RT-PCR and the ABI Prism 7900 HT Sequence Detection System (Applied Biosystems), and three independent experiments were used to quantify relative gene expression.

### Protein network analyses

The MS identified proteins which were confirmed to change in expression were further analyzed by Ingenuity Pathway Analysis (IPA) software (GeneGo Inc., St. Joseph, MI). IPA includes a manually annotated database of protein interactions and metabolic reactions obtained from the scientific literature. Gene names of all the identified proteins and the corresponding mean fold change values of the proteins were imported into IPA and processed using the shortest path algorithm. Hypothetical networks were built among the experimental proteins and the IPA database proteins. The relevant pathway maps were then prioritized according to their statistical significance (*p* < 0.001), and networks were graphically visualized as hubs (proteins) and edges (the relationship between proteins).

### Confocal microscopy

Cells (3 × 10^4^ per sample) were allow to adhere on glass coverslips at 37°C in humidified air with 5% CO_2_. After 24 hours, slides were washed with PBS, fixed and permeabilized with 2.5% formaldehyde-0.1% Triton X-100 in PBS for 10 minutes at 4°C. After several washes in PBS, slides were incubated with 10 μg/ml rabbit anti-FLNA polyclonal Ab (Cell Signaling) or 2 μg/ml R4 anti-uPAR mAb for 60 min at 23°C and then exposed to 1:700 goat Alexa Fluor 488 anti-rabbit IgG or goat Alexa Fluor 488 anti-mouse IgG, respectively (Molecular Probes) for 45 min at 23°C. To analyze cytoskeletal organization, coverslips were subsequently incubated with 0.1 μg/ml rhodamine-conjugated phalloidin (Invitrogen) at 23°C for 45 min. For uPAR-αv double staining, slides were incubated with 2 μg/ml anti-αv mAb (Chemicon, clone P3G8) and then with 1:800 goat Alexa Fluor 488 anti-mouse IgG (Molecular Probes) at 23°C for 60 and 45 minutes, respectively. Thereafter, 2 μg/ml R4 anti-uPARmAb and 1:800 diluted rabbit Alexa Fluor 594 anti-mouse IgG (Molecular Probes) were applied to slides at 23°C for 60 and 45 min, respectively. In all cases, slides were mounted using 20% (w/v) Mowiol, and cells were visualized with a 510 META-LSM confocal microscopy (Carl Zeiss).

### Invasion assay

Invasion assays were performed in Boyden chambers, using 8 μm pore size PVPF filters (Nucleopore) coated with 50 μg/filter matrigel (BD Biosciences ) as previously described [26]. Briefly, 3 × 10^4^ viable cells with/without 1:500 diluted blocking anti-αv monoclonal antibody, clone 272-17E6, (Merck Millipore, Darmstadt, Germany) or anti-Pan Akt (#MAB2055 R&D systems, Inc, Minneapolis, MN) monoclonal antibody, the last used as antibody negative control, were seeded in each upper chamber in serum-free DMEM. The lower chamber was filled with DMEM plus/minus 10% FBS as a source of chemo-attractants. Cells were allowed to invade matrigel for 18 hours at 37°C, 5% CO_2_. At the end of the assay, cells on the lower filter surface were fixed with ethanol, stained with haematoxylin and 10 random fields/filter were counted at 200x magnification. The arbitrary value of 100% was given to the basal cell invasion, assessed in the absence of serum. The experiments were performed three times in duplicate, and the results, expressed as percentage of the basal cell invasion.

### Oncomine gene expression analysis

Gene expression profiles of 2-DE DIGE validated proteins in prostate tumors were performed using the publically available Cancer Microarray Database Oncomine (www.oncomine.org).

### Statistical analysis

The RT-PCR data for mRNA expression are representative of at least three independent experiments and include the means ± S.D. of technical triplicates. Statistical significance was proved by two-sided Student's t-tests (normal distribution), and all statistically significant *P*-values (≤0.05) are reported in the manuscript or in figure legends. Representative results from Western blots from a single experiment are presented; additional experiments yielded similar results. All statistical evaluations were performed using Sigma Stat software (Systat Software Inc., San Jose, CA USA).

## SUPPLEMENTARY FIGURES AND TABLE


