# Supplementary material for: Proteomic analysis of zoledronic-acid resistant prostate cancer cells unveils novel pathways characterizing an invasive phenotype
Source: Oncotarget. 2014 Nov 24;6(7):5324–41. doi: 10.18632/oncotarget.2694 (PMC4467152; doi:10.18632/oncotarget.2694)
Supplement: Supplementary file 1 [file oncotarget-06-5324-s001.pdf]

SUPPLEMENTARY FIGURES AND TABLE

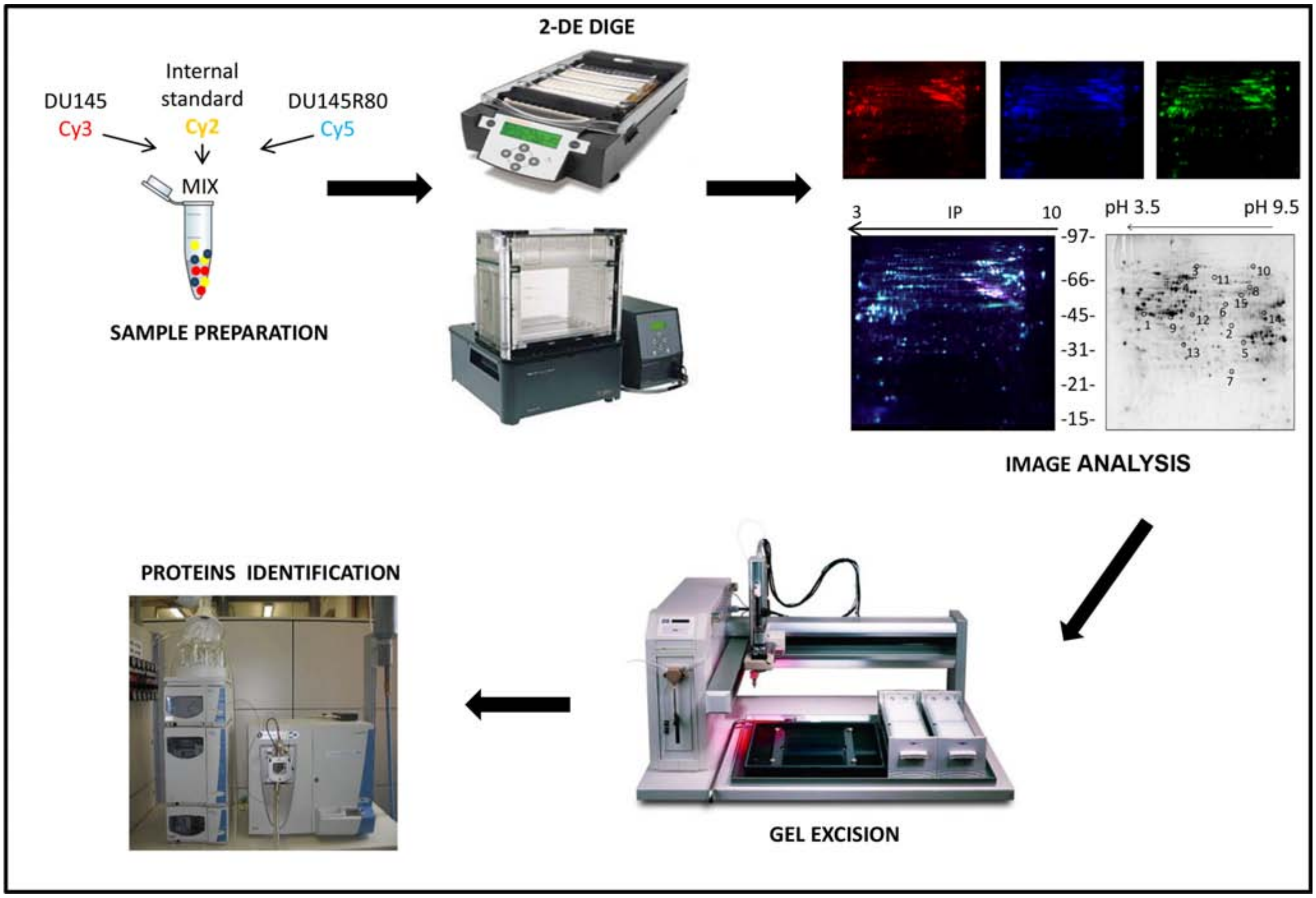

Supplementary Figure S1: 2-DE DIGE/MS workflow. Schematic representation of 2-DE DIGE/MS procedure from sample preparation and labeling to gel analysis and protein identification.

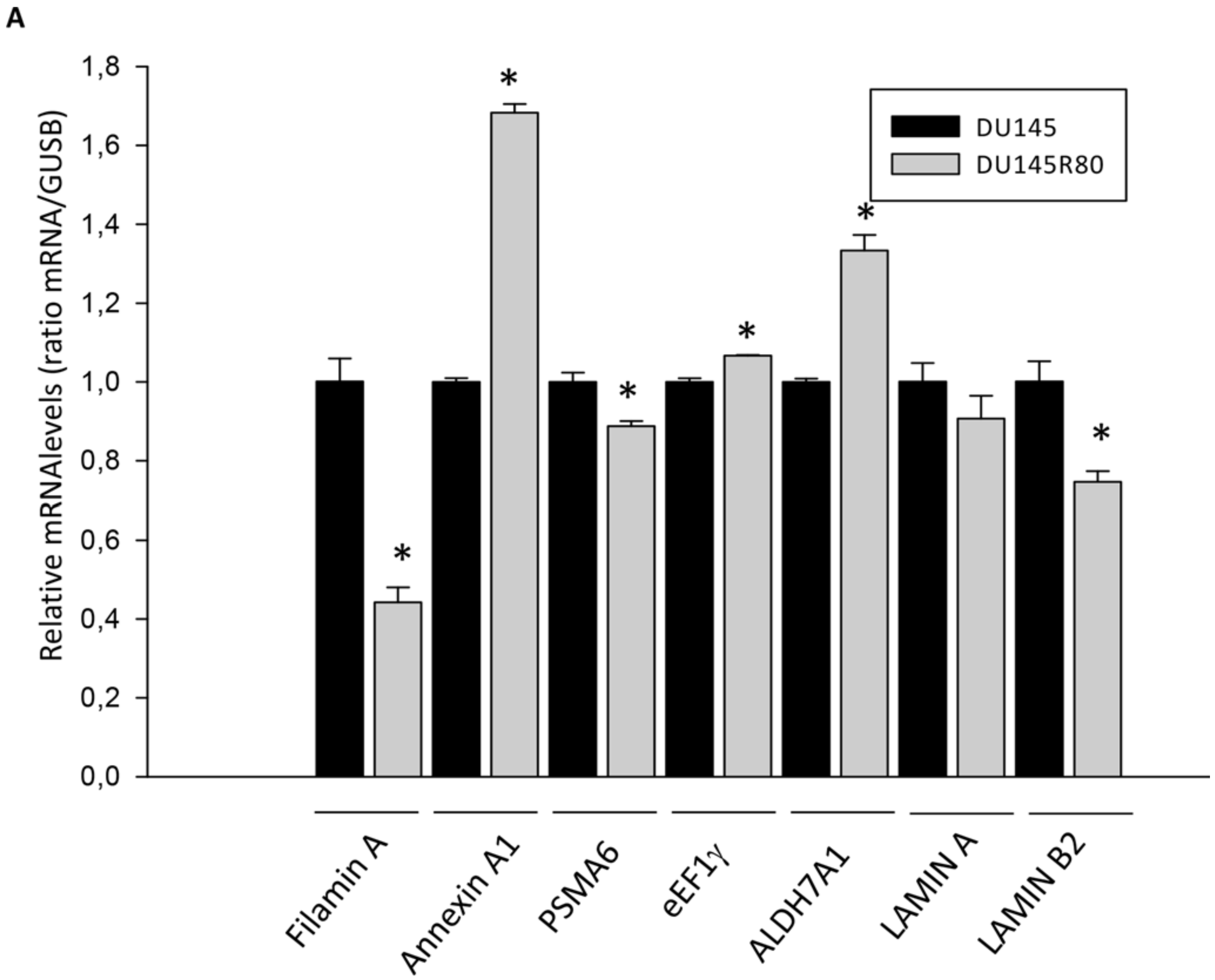

Supplementary Figure S2: Validation by Real Time PCR of protein identified as differentially expressed in the 2-D DIGE/MS analysis. FLMNA, ANXA1, PSMA6, eEF1γ, ALDH7A1, LMNA and LMNB2 mRNA expression was evaluated by Real-Time PCR after 24 h of cell culture. The data are representative of at least three independent experiments, include the means ± SD of technical triplicates and reported statistical analysis of DU145R80 versus DU145 cells (*P < 0.001, FLMNA; P < 0.001, ANXA1; P = 0.002, PSMA6; P = 0.003, eEF1γ; P < 0.001, ALDH7A1 ; P = 0.793, LMNA; P = 0,002, LMNB2).

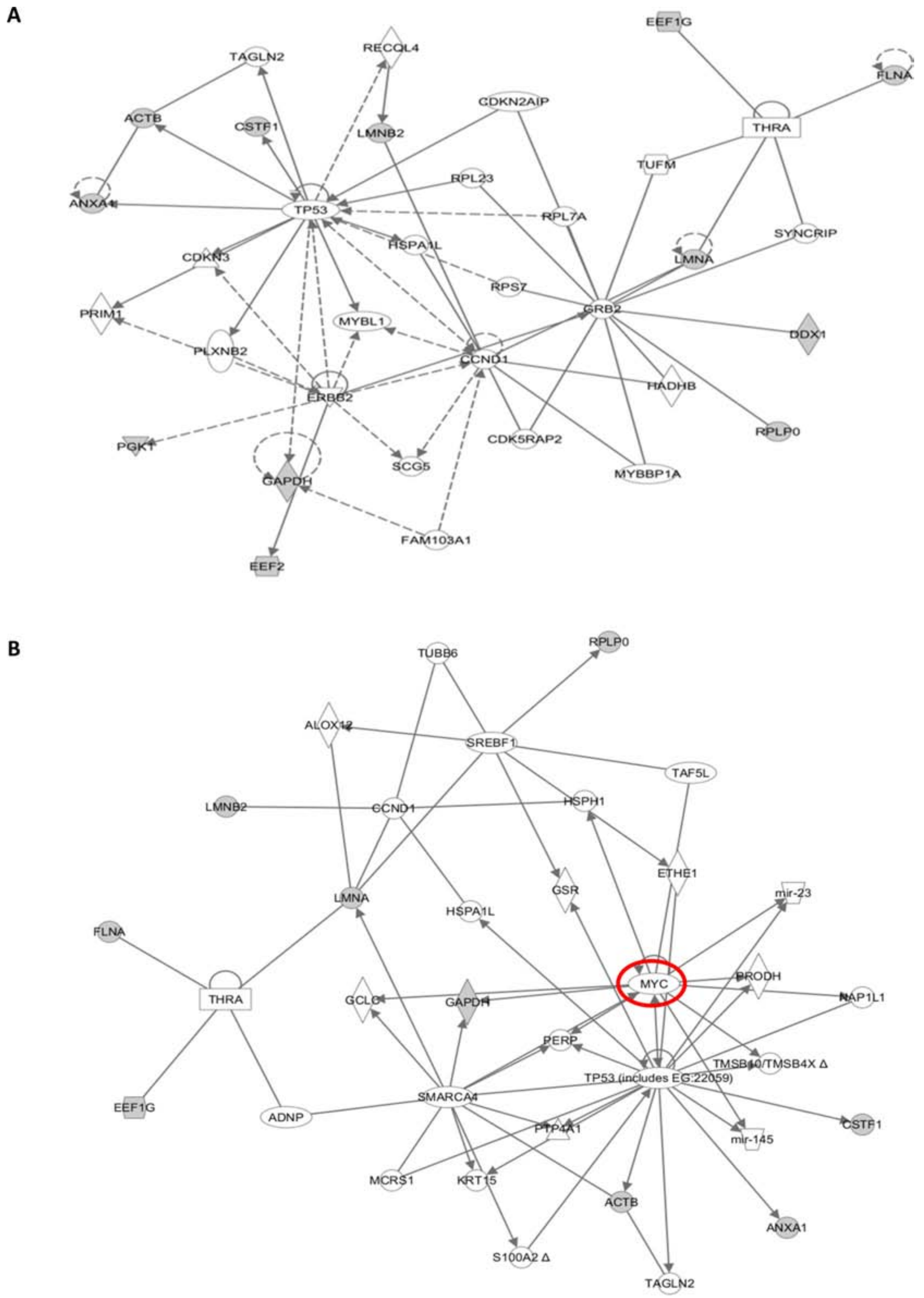

Supplementary Figure S3: IPA analysis. Visual representation of the principal network generated by Ingenuity Pathway Analysis (IPA). The network on the top (A, *P* = 1.02E^{-05}-44.31E^{-02} generated by including protein with direct and indirect relations contain 12 of the 15 proteins (80%) identified as differentially expressed in the 2-DE DIGE/MS analysis. The second network (B, *P* = 1.61E^{-05}-4.37E^{-02}) generated by including only protein with direct relations contain 9 of the 15 proteins (60%) identified as differentially expressed in the 2-DE DIGE/MS analysis. Network proteins are visualized by proper symbols, which specify the functional nature of protein. Each node represents a protein and its direct (represented by solid lines) and indirect (represented by dotted lines) association with other proteins. Proteins with no background colour were undetected in the study but have been inserted by IPA to produce a highly connected network, while the proteins colored in gray correspond to those which have been identified by 2-DE DIGE/MS analysis.

A

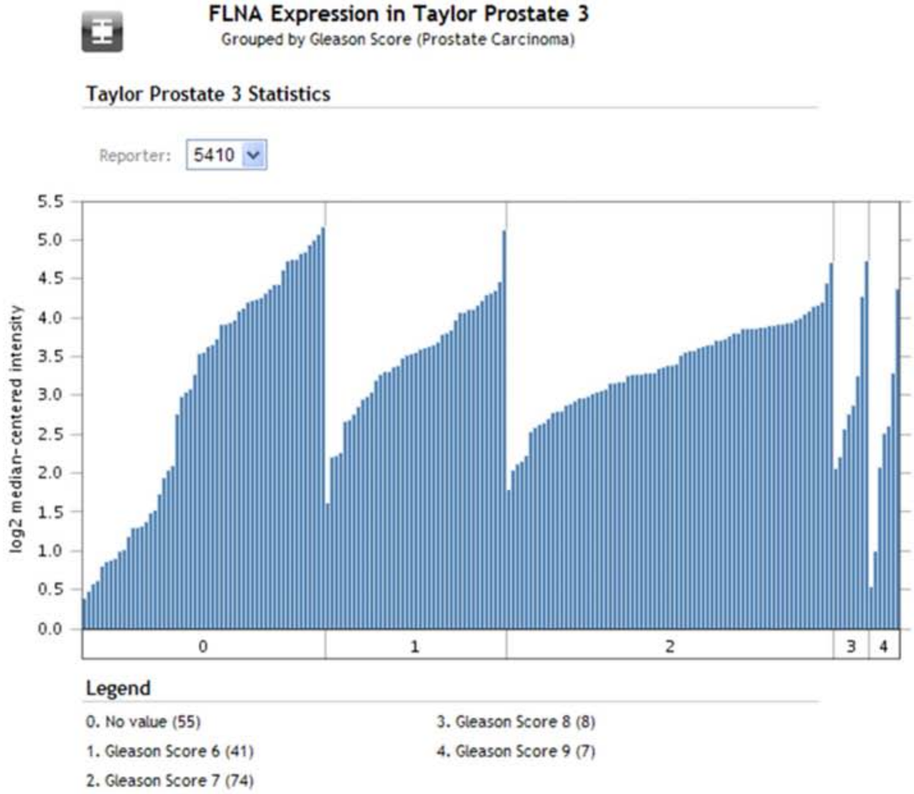

B

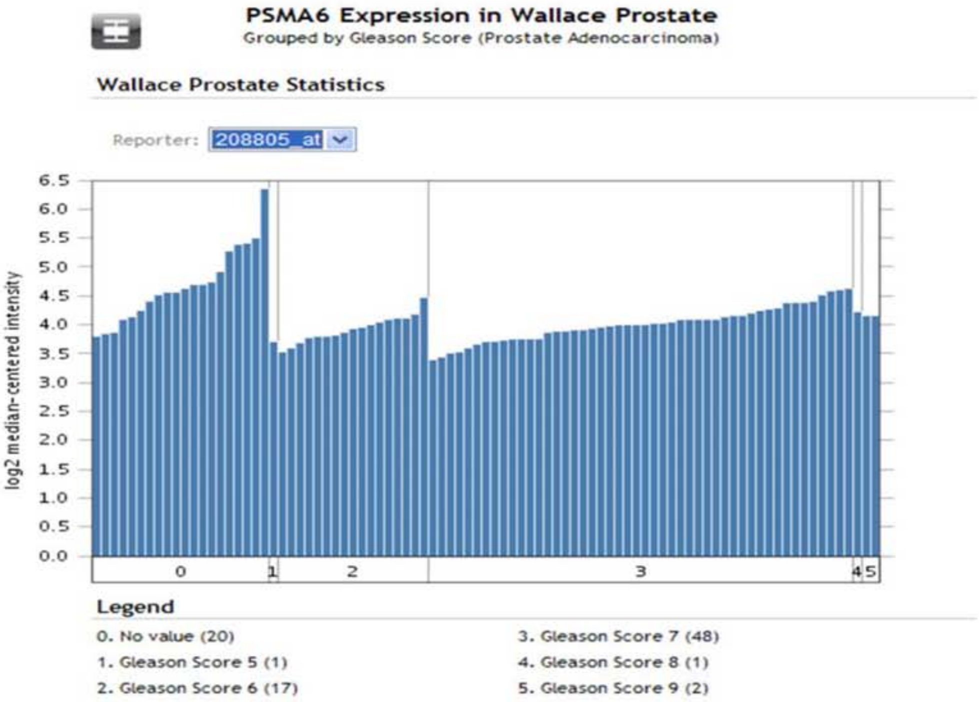

Supplementary Figure S4: Oncomine Data. mRNA expression of FLNA **(A)** and PSMA6 **(B)** in normal prostate gland and prostate carcinoma specimens from patients correlated with different gleason scores. Data were obtained from cDNA microarray analysis published in Oncomine database.

Supplementary Table S1. Experimental design for protein labeling

Gel Number	Cy2	Cy3	Cy5
1	Pooled Std*	DU145	DU145R80
2	Pooled Std*	DU145R80	DU145
3	Pooled Std*	DU145	DU145R80
4	Pooled Std*	DU145R80	DU145

Pooled Std*: pool served as an internal standard and consisted of equal amounts of each sample analyzed in 2-DE DIGE.
